# Clinicopathological features and prognostic significance of C5aR in human solid tumors: a Meta-analysis

**DOI:** 10.1186/s12885-021-08883-5

**Published:** 2021-10-23

**Authors:** Ziran Wang, Wenwei Yu, Yawen Qiang, Fan Ma, Pengsheng Ding, Yangyan Wang

**Affiliations:** 1 Department of Clinical Laboratory, Peking Union Medical College Hospital, Chinese Academy of Medical Sciences, Beijing, 100730 P. R. China; 2 Center of Reproductive Medicine, The First Affiliated Hospital of Anhui Medical University, Hefei, Anhui China; 3 Department of Obstetrics and Gynecology Laboratory, The First Affiliated Hospital of Anhui Medical University, Hefei, Anhui China; 4 Department of Clinical Laboratory, The Second Affiliated Hospital of Anhui Medical University, Hefei, Anhui China; 5 Department of Clinical Laboratory, The First Affiliated Hospital of USTC, Division of Life Sciences and Medicine, University of Science and Technology of China, Hefei, Anhui China; 6 Department of Clinical Laboratory, Yiji Shan Hospital, The First Affiliated Hospital of Wannan Medical College, Wuhu, Anhui China

**Keywords:** C5aR, Cancer, Prognosis, Clinicopathology, Meta-analysis

## Abstract

**Background:**

C5aR has been extensively studied in recent years as an essential component of the complement system. However, the role of C5aR in tumors has not been sufficiently investigated and summarized. The aim of this meta-analysis was to investigate the prognostic value of C5aR in solid tumors as well as the correlation between C5aR and clinicopathological features.

**Methods:**

Relevant study collection was performed in PubMed, Embase, Web of Science, BIOSIS Previews, Cochrane Library until July 10, 2021. Pooled hazard ratios (HRs), odds ratios (ORs), and 95% confidence intervals (CIs) were calculated. Sensitivity analyses were performed to assess the robustness of this study, while publication bias was tested by Begg’s and Egger’s tests.

**Results:**

A total of 11 studies involving 1577 patients were included in the study. Our results suggest that the high-level C5aR expression in tumor tissue predicted unsatisfactory overall survival (OS) (HR = 1.92, 95% CI:1.47–2.50, *P* < 0.001) and recurrence-free survival (RFS) (HR = 2.19, 95% CI:1.47–3.27, *P* < 0.001). Besides, a higher level of C5aR expression was associated with larger tumor size (OR = 1.58, 95% CI: 1.18–2.10, *P* = 0.002) and the occurrence of metastases in lymph nodes (OR = 1.99, 95% CI: 1.46–2.72, *P*<0.001), whereas it was independent of tumor stage, vascular invasion and tumor differentiation.

**Conclusion:**

In conclusion, C5aR may be a potential biomarker for evaluating tumor prognosis and treatment.

**Supplementary Information:**

The online version contains supplementary material available at 10.1186/s12885-021-08883-5.

## Background

Cancers have become a major global public health problem, bringing a heavy burden to society. In 2020, there were approximately 19.3 million new cancer cases and 10.0 million cancer deaths worldwide [1]. The treatment of cancers has come a long way from the traditional surgical resection, radiotherapy and chemotherapy to the recently developed immune checkpoint therapy [2]. Frustratingly, despite enormous progress being made in terms of cancer treatment, cancers are still the leading cause of death. Meanwhile, we also noted that cancer treatment is highly varied among individuals and that the prognosis varies significantly from one individual to another [3]. Therefore, in the background of personalized cancer treatment and assessment, a biomarker capable of predicting the clinicopathological features and prognosis of cancers is desired.

The complement system plays an essential role in immune regulation as it is involved in the pathological processes of inflammation and immune diseases as well as in the adaptive immune response, in addition to being involved in host defense mechanisms [4]. The complement component C5a is a potent pro-inflammatory factor that is associated with a wide range of diseases [5, 6]. C5a can bind two receptors, known as C5aR (CD88) and C5L2 (GPR77). C5aR is expressed at substantially higher levels on immune and non-immune cells than C5L2, and it is now thought that C5a exerts its functional effects mainly through C5aR [7]. Upregulation of C5aR expression has been proven to be implicated in the progression of many immune and inflammatory diseases, such as systemic lupus erythematosus [8], inflammatory bowel diseases [9], sepsis [10], and respiratory distress syndrome [11]. Furthermore, C5aR was reported to be overexpressed in a variety of tumors, including non-small cell lung cancer (NSCLC) [12–14], gastric cancer (GC) [15, 16], hepatocellular carcinoma (HCC) [17], urothelial cell carcinoma (UCC) [18], prostate cancer (PC) [19], renal cell carcinoma (RCC) [20, 21], and breast cancer (BC) [22]. However, the prognostic value of C5aR in cancers has not been fully elucidated.

The aim of this meta-analysis was to investigate the prognostic value of C5aR in solid tumors as well as the correlation between C5aR and clinicopathological features.

## Methods

### Search strategy

Our study was conducted in compliance with the Preferred Reporting Items for Systematic Reviews and Meta-Analyses (PRISMA) guidelines [23] and has been registered on the PROSPERO website (registration number, CRD42020191587). The PRISMA checklist is shown in Table S1. A total of five electronic databases (PubMed, Embase, Web of Science, BIOSIS Previews, Cochrane Library) were available for searching the literature, updated to July 10, 2021. The keywords used in the search were: (“C5aR” OR “C5a Receptor” OR “complement component C5a Receptor” OR “CD88”) AND (“neoplasms” OR “cancer” OR “tumor” OR “carcinoma” OR “leukemia” OR “lymphoma”). Moreover, references of retrieved articles were manually screened for including potential eligible literature.

### Inclusion and exclusion criteria

Literature was considered eligible when it fulfilled the following criteria: (1) Articles investigated the correlation of C5aR expression with patient prognosis and/or tumor clinicopathological features. (2) C5aR expression was measured and cancer patients were classified into high and low expression groups. (3) Articles provided HRs, ORs, and 95% CIs, or provided sufficient data to calculate them. (4) Articles were published in English. The exclusion criteria were as follows: (1) Duplicated publications. (2) Reviews, case reports, letters, conference abstracts. (3) Related studies were conducted in cell lines or at the animal level. (4) Insufficient data to calculate effect sizes.

### Data extract

Three investigators (ZRW, WWY and YWQ) screened the literature and extracted data from it independently, with any discrepancies resolved by consultation. Relevant data extracted include name of the first author, date of publication, country, tumor type, sample size, detection method, clinicopathological characteristics, OS, RFS, and 95% CI. If the prognostic data were presented as a Kaplan-Meier curve only, the Engauge Digitizer (version 4.1) software was used to calculate the HR and 95% CI as described [24].

### Quality assessment

The quality of the included literature was assessed according to the Newcastle-Ottawa Scale (NOS) criteria [25]. NOS scores are assigned on a scale of 0–9, with studies scoring≥6 being considered to be of high quality.

### Data analysis

All statistical analyses were performed using STATA software (version 14.0, Stata Corporation, College Station, TX, USA). OS and RFS were assessed by pooled HRs and 95% CIs. Clinicopathological features were assessed by pooled ORs and 95% CIs. Heterogeneity of all enrolled literature was assessed using χ^2^-based Q test and I^2^ statistics. A fixed-effects model was used where there was no heterogeneity (*P* > 0.1 and *I*^*2*^ < 50%), otherwise a random-effects model would have been applied. Funnel plots were used to estimate publication bias by visual inspection, and Begg’s and Egger’s tests were used to assess publication bias quantitatively, with *P* < 0.05 considered to be the presence of publication bias. Robustness of the meta-analysis was determined by removing studies one by one.

## Results

### Literature information

**Fig. 1:**
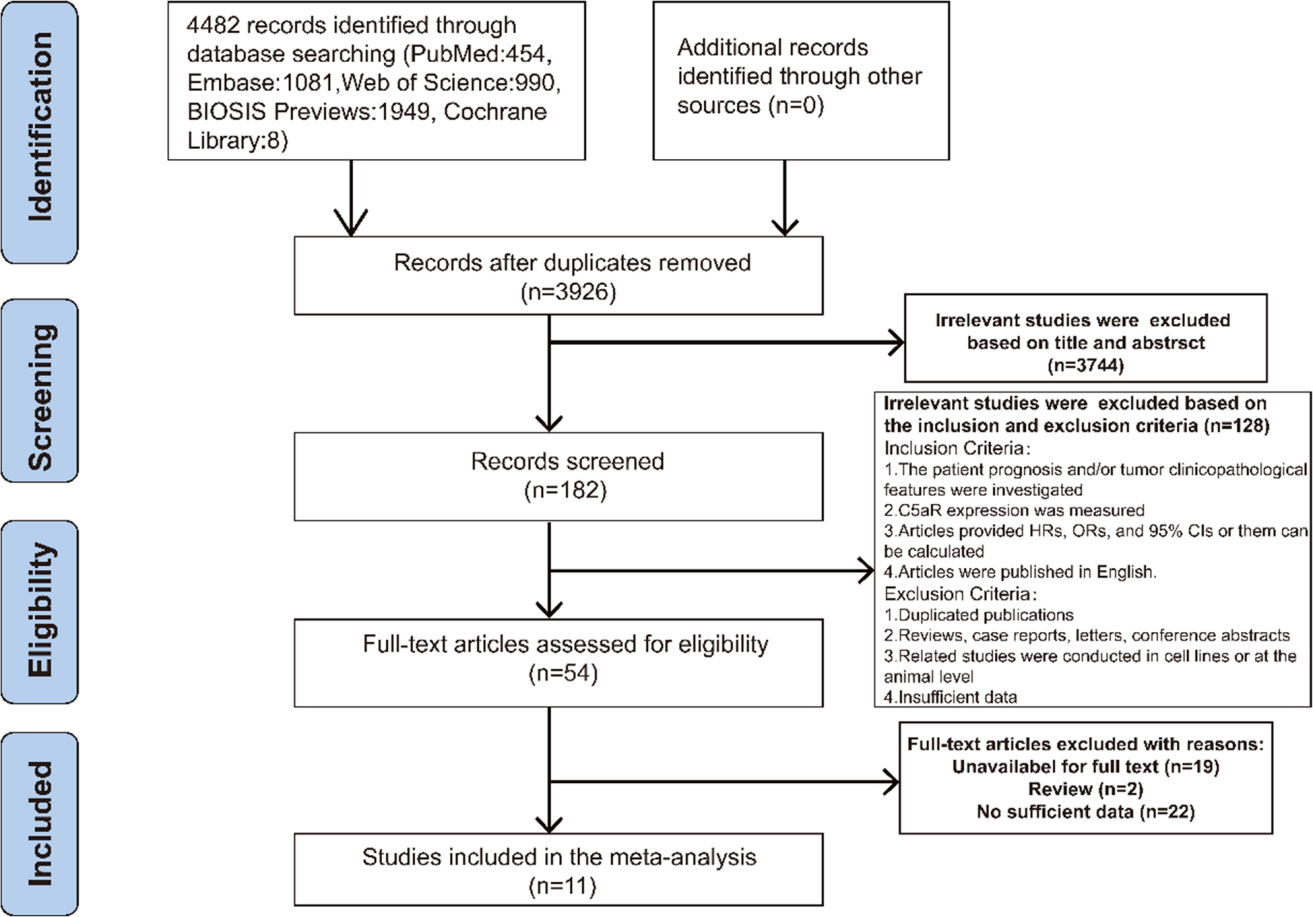
Flow diagram of the literature search in this meta-analysis

As depicted in Fig. 1, a total of 4482 records were retrieved by searching five databases (PubMed, Embase, Web of Science, BIOSIS Previews, Cochrane Library). After removing duplicate papers, 3926 records remained. Furthermore, 182 studies were eligible for initial screening based on title and abstract. According to the inclusion and exclusion criteria we established, 128 irrelevant studies were excluded. The remaining 54 studies were reviewed for full text, with the final 11 studies meeting the requirements.

### Study characteristics

The clinical characteristics of the included studies were summarized in Table S2. These 11 studies comprised a total of 1577 patients from 2013 to 2020. Almost all of these studies came from China or Japan, while only one study came from Spain. Cancer Type contains NSCLC, GC, HCC, UCC, PC, RCC and BC. Seven articles investigated the relationship between C5aR expression and OS, including a total of 1026 patients. Besides, four articles containing 666 cases studied the correlation between C5aR expression and RFS. Ten studies with a total of 1502 patients focused on the correlation between C5aR expression and clinicopathological features. All eligible papers have a high quality with NOS scores in the range of 7–9.

### C5aR expression and OS

**Fig. 2:**
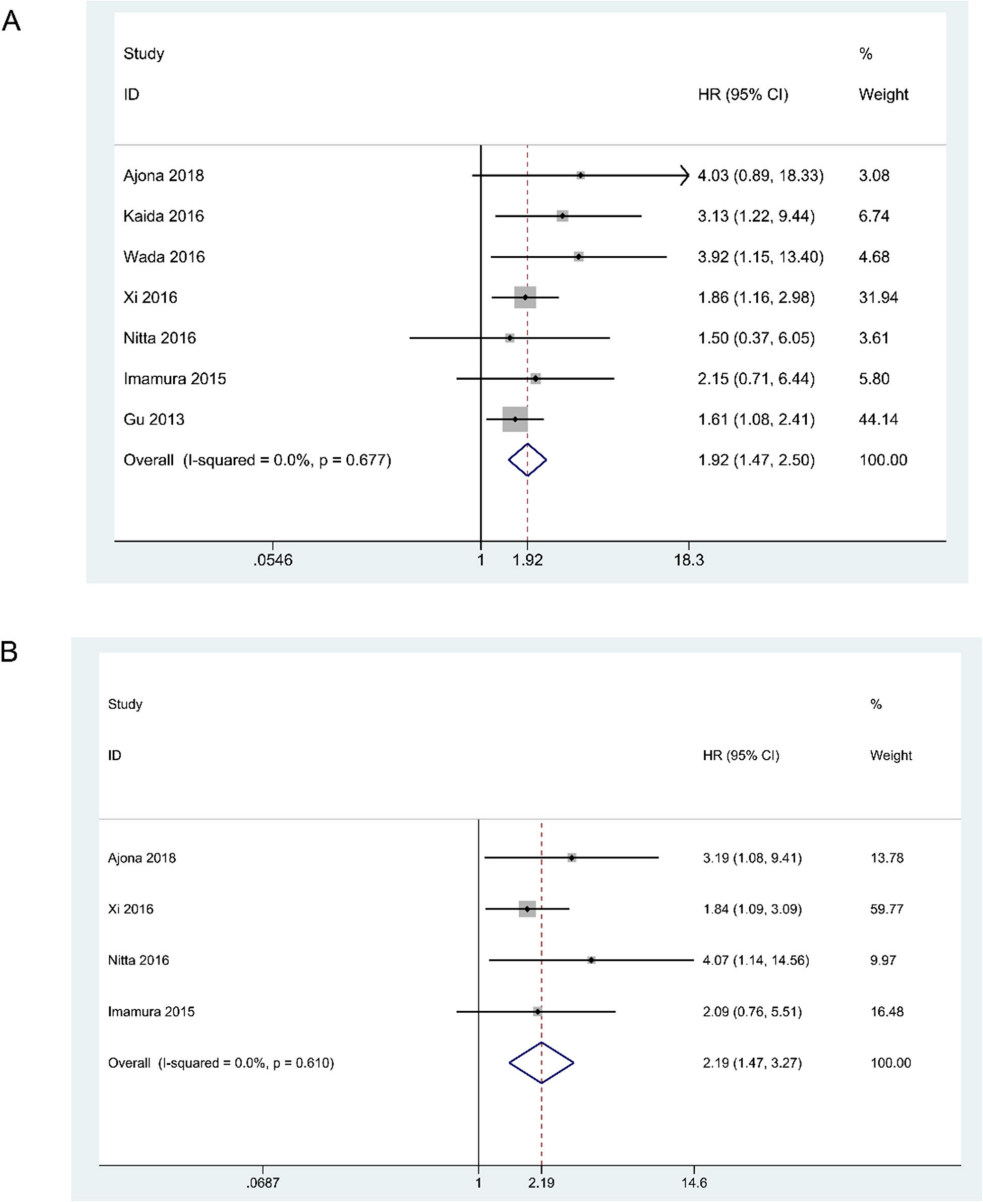
Forest plot of studies evaluating the associations between the C5aR expression levels and prognostic indicators. A, overall survival (OS); B, recurrence-free survival (RFS)

Seven studies were conducted to explore the relationship between C5aR and prognosis in terms of OS, with detailed information in Table 1. Since there was no heterogeneity (*I*^*2*^ = 0.0%, *P* = 0.677), the fixed-effects model was used to pool the data. The pooled results indicate that higher C5aR expression was associated with a poorer prognosis (HR = 1.92, 95% CI:1.47–2.50, *P* < 0.001). The forest plot was shown in Fig. 2A.

**Table 1:** Characteristics of eligible studies for prognosis

Study	Year	Country	Tumor type	Sample size	Detection method	Survival analysis	Analysis type	Source of HR	NOS score
Ajona et al.	2018	Spain	NSCLC	75	IHC	OS, RFS	Multivariate	Reported	8
Kaida et al.	2016	Japan	GC	100	IHC	OS	Multivariate	Reported	8
Wada et al.	2016	Japan	UCC	52	IHC	OS	Multivariate	Reported	7
Xi et al.	2016	China	RCC	272	IHC	OS, RFS	Multivariate	Reported	9
Nitta et al.	2016	Japan	GC	148	IHC	OS, RFS	Multivariate	Survival Curve	9
Imamura et al.	2015	Japan	BC	171	IHC	OS, RFS	Multivariate	Reported	8
Gu et al.	2013	China	NSCLC	208	IHC	OS	Multivariate	Reported	8

*NSCLC* Non-small cell lung cancer, *GC* Gastric cancer, *HCC* Hepatocellular carcinoma, *UCC* Urothelial cell carcinoma, *RCC* Renal cell carcinoma, *BC* Breast cancer, *IHC* Immunohistochemistry, *HR* Hazard ratio, *NOS* Newcastle-Ottawa Scale, *OS* Overall survival, *RFS* Recurrence-free survival

### C5aR expression and RFS

Four studies assessed RFS in patients with different levels of C5aR expression **(**Table 1**)**. We used a fixed-effects model due to the lack of heterogeneity (*I*^*2*^ = 0.0%, *P* = 0.610). As illustrated in Fig. 2B, a higher level of C5aR predicted that the patient had an undesirable RFS. (HR = 2.19, 95% CI:1.47–3.27, *P* < 0.001).

### C5aR expression and clinicopathological features

We systematically investigated the correlation between C5aR expression and clinicopathological features, including tumor size, lymph node metastasis, tumor stage, vascular invasion and differentiation. The results were summarized in Tables 2 and 3. We found that a higher C5aR level was positively correlated with tumor size (OR = 1.58, 95%CI: 1.18–2.10, *P* = 0.002) and lymph node metastasis (OR = 1.99, 95%CI: 1.46–2.72, *P*<0.001) (Fig. S1). However, C5aR expression did not show a significant correlation with tumor stage (OR = 1.47, 95%CI: 0.93–2.34, *P* = 0.102), vascular invasion (OR = 1.66, 95%CI: 0.55–5.01, *P* = 0.368) and tumor differentiation (OR = 1.10, 95%CI: 0.74–1.62, *P* = 0.646). We noted that for tumor stage, there was a large heterogeneity (*I*^*2*^ = 65.5%, *P* = 0.002). Therefore, we implemented the subgroup analysis based on sample size and the results showed that for sample size≥100, higher C5aR expression was more prone to develop advanced tumor stage (OR = 1.88, 95%CI: 1.30–2.71, *P* = 0.001) with a low heterogeneity (*I*^*2*^ = 39.5%, *P* = 0.116) (Fig. S1). However, for the two studies with sample size<100, the combined data reached the opposite conclusion (OR = 0.38, 95%CI: 0.18–0.81, *P* = 0.924) with heterogeneity. Thus, sample size may be a source of heterogeneity.

**Table 2:** Characteristics of eligible studies for clinicopathological features

Study	Year	Country	Tumor type	Samplesize	Male/Female	High/Low C5aR	Clinicopathologic Features
Zhao et al.	2018	China	NSCLC	185	128/57	104/81	Tumor size, Lymph node metastasis, TNM stage, Pathologic type
Kaida et al.	2016	Japan	GC	100	64/36	35/65	Tumor location, Differentiation, Depth of invasion, Lymph node metastasis, pStage, Lymphatic invasion, Vascular invasion
Hu et al.	2016	China	HCC	78	51/27	53/25	Tumor size, Tumor numbers, Capsular invasion, E-cadherin expression, Snail expression, Claudin-1 expression, Pathological grade, Tumor stage
Wada et al.	2016	Japan	UCC	52	39/13	38/14	Tumor location, WHO grade, T stage, Blood vessel invasion, Lymph node invasion, Stage of disease
Imamura et al.	2020	Japan	PC	161	NA	32/129	Gleason grade, Pathological Tstage, PD-L1 expression
Maeda et al.	2015	Japan	RCC	127	86/41	78/49	Histological subtypes, Fuhrman grade, TNM stage, microscopic invasion
Xi et al.	2016	China	RCC	272	188/84	141/131	Tumor size, Fuhrman grade, Necrosis, TNM stage, ECOG-PS
Nitta et al.	2016	Japan	GC	148	108/40	45/103	Tumor size, Location, Differentiation, Invasion depth, N classification, pStage, Lymphatic invasion, Vascular invasion, Amount of interstitial connective tissue, Infiltrative pattern
Imamura et al.	2015	Japan	BC	171	0/171	22/149	Menopause, Pathological tumor size, nuclear grade, Ki-67 labeling index, Nodular status, Clinical stages, Estrogen receptor (ER), Estrogen receptor (ER), HRE2, Tumor subtype
Gu et al.	2013	China	NSCLC	208	148/60	111/97	Smoking status, Histological type, Tumor stage, Lymph node metastasis, Tumor size, Differentiation

*NSCLC* Non-small cell lung cancer, *GC* Gastric cancer, *HCC* Hepatocellular carcinoma, *UCC* Urothelial cell carcinoma, *RCC* Renal cell carcinoma, *BC* Breast cancer

**Table 3:** Meta-analysis results for C5aR expression with clinicopathological features

Clinicopathologic features	No. of studies	No. of patients	Estimate OR (95% CI)	***P*** value	***I***^***2***^ (%)	***P*** value	Model
**Tumor size (big vs. small)**	**5**	**891**	**1.58 (1.18, 2.10)**	**0.002**	**47.70%**	**0.105**	**Fixed**
**Lymph node metastasis (yes vs. no)**	**6**	**820**	**1.99 (1.46, 2.72)**	**<0.001**	**27.30%**	**0.230**	**Fixed**
Tumor stage (III-IV vs. I-II)	10	1502	1.47 (0.93, 2.34)	0.102	65.50%	0.002	Random
Vascular invasion (yes vs. no)	5	505	1.66 (0.55, 5.01)	0.368	84.00%	<0.001	Random
Tumor differentiation (well vs. poor)	3	456	1.10 (0.74, 1.62)	0.646	18.00%	0.295	Fixed

*No.* Number; *OR* Odds ratio, *CI* Confidence interval

The results are in bold if *P* < 0.05

### Sensitivity analysis

**Fig. 3:**
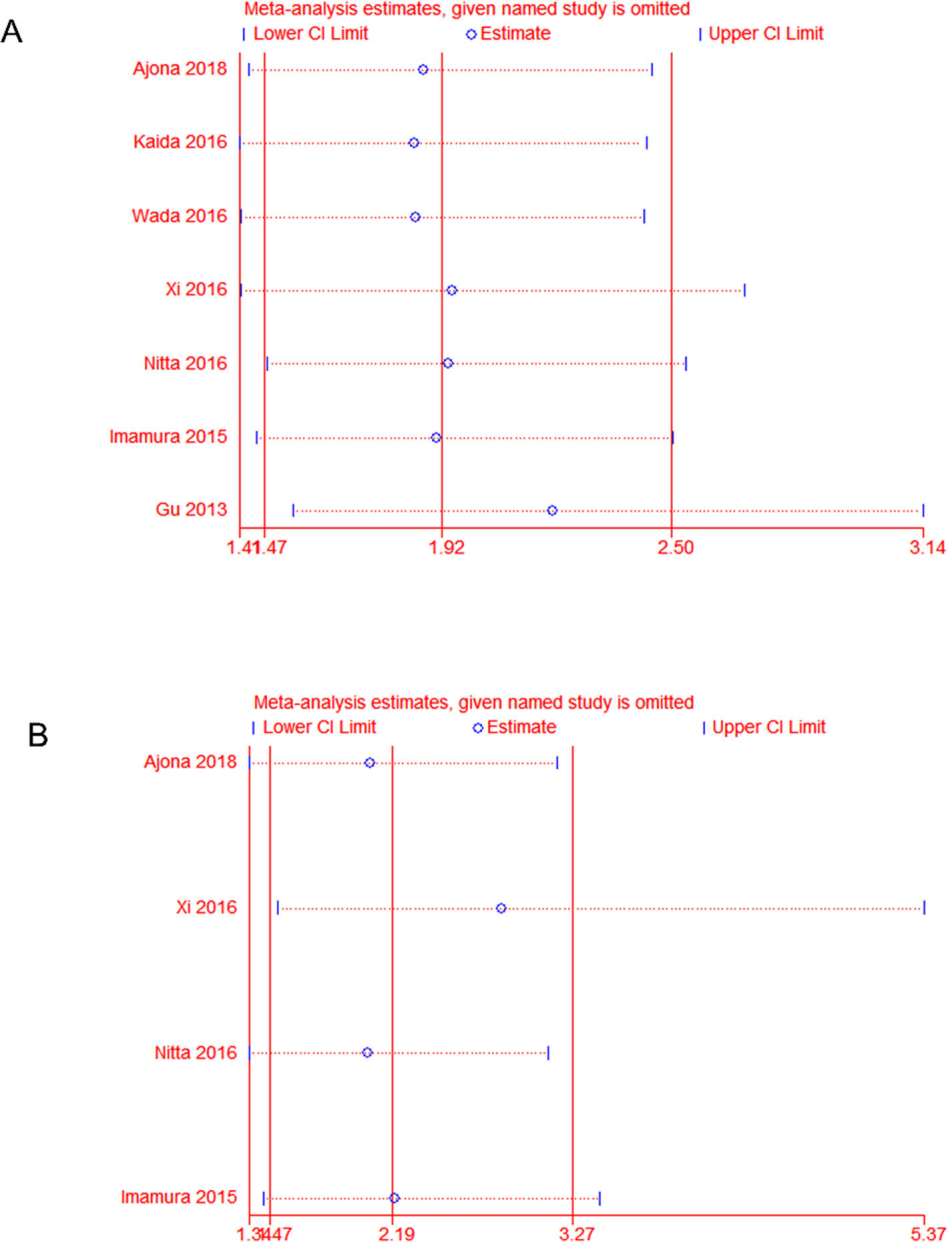
Sensitivity analysis of studies evaluating the associations between the C5aR expression levels and prognostic indicators. A, overall survival (OS); B, recurrence-free survival (RFS)

Sensitivity analysis was performed to check the stability of the results by removing the studies one by one. As shown in Fig. 3 and Fig. S2, removing either study did not have a dramatic effect on the pooled values of OS, RFS and clinicopathological characteristics.

### Publication Bias

**Fig. 4:**
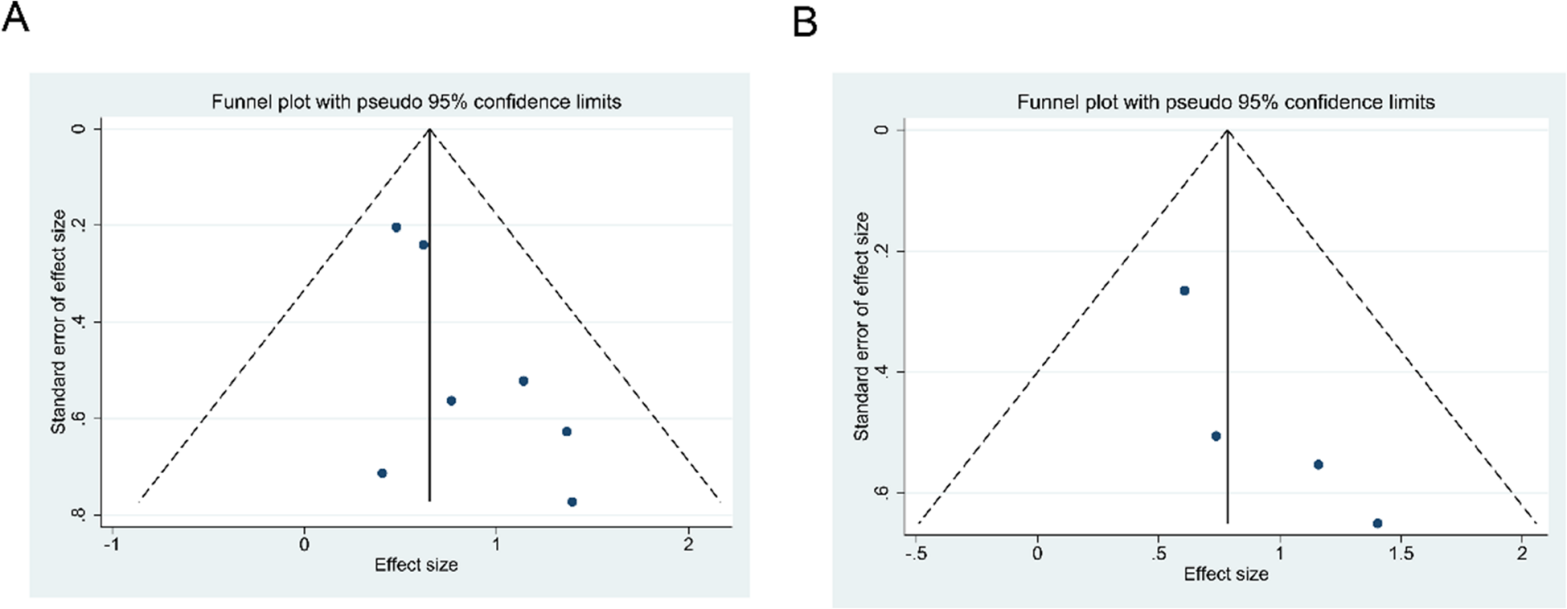
Funnel plot for publication bias in this meta-analysis. A, overall survival (OS); B, recurrence-free survival (RFS)

Both the Begg’s and Egger’s tests were used to assess potential publication bias. The results show that the *P* value>0.05 for OS, RFS and clinicopathological characteristics (Table 4), implying that there was no publication bias in this meta-analysis. Besides, the large symmetry of the funnel plot from a visual perspective again validated the absence of publication bias (Fig. 4 and Fig. S3).

**Table 4:** Summary of publication bias tests in this meta-analysis

Parameters	Begg’s test ***P*** value	Egger’s test ***P*** value
OS	0.230	0.057
RFS	0.089	0.102
Tumor size	0.462	0.695
Lymph node metastasis	0.707	0.801
Tumor stage	1.000	0.855
Vascular invasion	0.806	0.993
Tumor differentiation	1.000	0.530

*OS* Overall survival, *RFS* Recurrence-free survival

## Discussion

C5aR has garnered rising interests in recent years as an important component of the immune regulatory system. In sepsis, excessive activation of C5a-C5aR signaling leads to functional paralysis of neutrophils, and blocking C5a or C5aR can effectively improve survival in septic patients [26]. Inhibition of the activation of the C5a-C5aR signaling pathway can inhibit the inflammatory response early and thus reverse the transition from acute kidney injury to renal failure [27]. Notably, therapy targeting C5aR in non-immune cells can reduce inflammation and tissue damage in the lung, bringing a new light to the treatment of COVID-19 [28]. A variety of inhibitors targeting C5aR have been proven to reduce the detrimental effects of inflammatory diseases [29, 30]. Unfortunately, the role of C5aR in cancers has not been systematically studied and summarized. In our previous study, we demonstrated that the S component of Panton-Valentine leucocidin (LukS-PV) can effectively inhibit the progression of hepatocellular carcinoma cells with a higher expression of C5aR, revealing that C5aR may be an important target for cancer therapy [31].

In this meta-analysis, we comprehensively investigated the correlation between C5aR expression and solid tumors prognosis as well as clinicopathological features. Our results suggest that the high-level C5aR expression in tumor tissue predicted unsatisfactory OS (HR = 1.92, 95% CI:1.47–2.50, *P* < 0.001) and RFS (HR = 2.19, 95% CI:1.47–3.27, *P* < 0.001). Hence, C5aR may be an excellent indicator for evaluating tumor prognosis. Subsequently, a higher level of C5aR expression was associated with larger tumor size (OR = 1.58, 95%CI: 1.18–2.10, *P* = 0.002) and the occurrence of metastases in lymph nodes (OR = 1.99, 95%CI: 1.46–2.72, *P*<0.001), whereas it was independent of tumor stage, vascular invasion and tumor differentiation. Intriguingly, there was an apparent heterogeneity in the analysis of tumor stage, but subgroup analysis based on sample size drew the contrary conclusions. It can thus be seen that the sample size was determinant for the final conclusions. In the future, more studies with larger samples would be helpful to further clarify the relationship between C5aR and tumor stage.

In terms of mechanism, a growing body of evidence highlights the crucial role of C5aR in tumor progression. It has been reported that blocking C5aR inhibited the progression of breast cancer through the p38/p21 signaling axis [32] . Hu et al. reported that C5aR promoted hepatocellular carcinoma cell invasion and metastasis through ERK1/2-mediated epithelial mesenchymal transition (EMT) [17]. In addition, the administration of PD-1/PD-L1 antibodies enabled the hyperactivation of the C5a-C5aR pathway, PD-1/PD-L1 antibodies combined with C5aR blockade therapy could achieve a satisfactory anti-tumor effect [33]. C5aR can also facilitate tumor metastasis by suppressing the response of CD4^+^ and CD8^+^ T cells in the lung, possibly driven by the recruitment of immature myeloid cells to the lungs and the production of large amounts of TGF-β and IL10 [34]. Given the interactive role of C5aR in cancer signaling pathways and tumor immunity, therapies targeting C5aR are promising directions to be developed in the future.

Certainly, there were some limitations to this study. Firstly, our study should be regarded as preliminary because a small number of articles included in this meta-analysis, especially regarding prognosis. In addition, inadequate data may limit the accuracy and validity of the conclusions of this study. We also look forward to more high-quality studies involving the assessment of prognostic and clinicopathological features of C5aR in cancers. Secondly, some of the studies only had survival curves as an indicator of prognosis, and we had to use software to estimate HRs and 95% confidence intervals, which may have deviated from the true values. Thirdly, almost all of the patients included in the study were from China and Japan, and studies covering other countries and races were scarce. Finally, studies on the treatment analysis with C5aR expression were missing.

## Conclusion

In summary, our meta-analysis reveals that a higher level of C5aR expression was associated with poorer prognosis, larger tumor size and the development of lymph node metastases. Therefore, C5aR may be a potential biomarker for evaluating tumor prognosis and treatment.

## Supplementary Information

**Additional file 1 **: **Table S1.** PRISMA 2020 Checklist of this study.

**Additional file 2: Table S2**. The clinical characteristics of the included studies. **Fig. S1.** Forest plot of studies evaluating the associations between the C5aR expression levels and clinicopathological features. **Fig. S2.** Sensitivity analysis of studies evaluating the associations between the C5aR expression levels and clinicopathological features. **Fig. S3.** Funnel plot for publication bias in this meta-analysis.

## Data Availability

The data that support the findings of this study are available on request from the corresponding author.

## Abbreviations

C5aR: C5a receptor
OS: Overall survival
RFS: Recurrence-free survival
HR: Hazard ratio
OR: Odds ratio
CI: Confidence interval
IHC: Immunohistochemistry
NSCLC: non-small cell lung cancer
GC: gastric cancer
HCC: hepatocellular carcinoma
UCC: urothelial cell carcinoma
RCC: renal cell carcinoma
BC: breast cancer
