# Supplementary material for: Clinicopathological features and prognostic significance of C5aR in human solid tumors: a Meta-analysis
Source: BMC Cancer. 2021 Oct 23;21:1136. doi: 10.1186/s12885-021-08883-5 (PMC8540875; doi:10.1186/s12885-021-08883-5)
Supplement: Supplementary file 2 — Additional file 2: Table S2. The clinical characteristics of the included studies. Fig. S1. Forest plot of studies evaluating the associations between the C5aR expression levels and clinicopathological features. Fig. S2. Sensitivity analysis of studies evaluating the associations between the C5aR expression levels and clinicopathological features. Fig. S3. Funnel plot for publication bias in this meta-analysis. [file 12885_2021_8883_MOESM2_ESM.docx]

**Table S2** The clinical characteristics of the included studies

| **No.** | **Year** | **First author** | **Country** | **Cancer type** | **Sample size** | **Method** | **Male/Female** | **High/Low C5aR** | **OS** | **RFS** | **Survival analysis** | **Source of HR** | **NOS** | **Clinical characteristics** |
| --- | --- | --- | --- | --- | --- | --- | --- | --- | --- | --- | --- | --- | --- | --- |
| 1 | 2018 | Daniel Ajona | Spain | NSCLC | 75 | IHC | 66/9 | 50/25 | 4.032 (0.887-18.325) | 3.187 (1.079-9.411) | MU | Reported | 8 | NA |
| 2 | 2018 | Chenhui Zhao | China | NSCLC | 185 | IHC | 128/57 | 104/81 | NA | NA | NA | NA | 8 | Tumor size, Lymph node metastasis, TNM stage, Pathologic type |
| 3 | 2016 | Takayoshi Kaida | Japan | GC | 100 | IHC | 64/36 | 35/65 | 3.13 (1.12-9.44) | NA | MU | Reported | 8 | Tumor location, Differentiation, Depth of invasion, Lymph node metastasis, Lymph node metastasis, Lymphatic invasion, Vascular invasion |
| 4 | 2016 | Wenhao Hu | China | HCC | 78 | IHC | 51/27 | 53/25 | NA | NA | NA | NA | 8 | Tumor size, Tumor numbers, Capsular invasion, E-cadherin expression, Snail expression, Claudin-1 expression, Pathological grade, Tumor stage |
| 5 | 2016 | Yoshihiro Wada | Japan | UCC | 52 | IHC | 39/13 | 38/14 | 3.92 (1.15‑13.4) | NA | MU | Reported | 7 | Tumor location, WHO grade, T stage, Blood vessel invasion, Lymph node invasion, Stage of disease |
| 6 | 2020 | Ryuji Imamura | Japan | PC | 161 | IHC | NA | 32/129 | NA | NA | NA | NA | 7 | Gleason grade, Pathological Tstage, PD‐L1 expression |
| 7 | 2015 | Yoshihiro Maeda | Japan | RCC | 127 | IHC | 86/41 | 78/49 | NA | NA | NA | NA | 8 | Histological subtypes, Fuhrman grade, TNM stage, Microscopic invasion |
| 8 | 2016 | Wei Xi | China | RCC | 272 | IHC | 188/84 | 141/131 | 1.860 (1.163–2.977) | 1.835 (1.091–3.087) | MU | Reported | 9 | Tumor size. Fuhrman grade, Necrosis, TNM stage, ECOG-PS |
| 9 | 2016 | Hidetoshi Nitta | Japan | GC | 148 | IHC | 108/40 | 45/103 | 1.5 (0.37-6.05) | 4.07 (1.14-14.56) | NA | SC | 9 | Tumor size, Location, Differentiation, Invasion depth, N classification, pStage, Lymphatic invasion, Vascular invasion, Amount of interstitial connective tissue, Infiltrative pattern |
| 10 | 2015 | Takahisa Imamura | Japan | BC | 171 | IHC | 0/171 | 22/149 | 2.15 (0.71-6.44) | 2.09 (0.76-5.51) | MU | Reported | 8 | Menopause, Pathological tumor size, nuclear grade, Ki-67 labeling index, Nodular status, Clinical stages, Estrogen receptor (ER), Estrogen receptor (ER), HRE2, Tumor subtype |
| 11 | 2013 | Jie Gu | China | NSCLC | 208 | IHC | 148/60 | 111/97 | 1.614 (1.082-2.407) | NA | MU | Reported | 8 | Smoking status, Histological type, Tumor stage, Lymph node metastasis, Tumor size, Differentiation |

NSCLC, non-small cell lung cancer; GC, gastric cancer; HCC, hepatocellular carcinoma; UCC, urothelial cell carcinoma; PC prostate cancer; RCC, renal cell carcinoma; BC, breast cancer; HR, Hazard ratio; NOS, Newcastle-Ottawa Scale; OS, Overall survival; RFS, Recurrence-free survival; MU, multivariate analysis; NA, not available


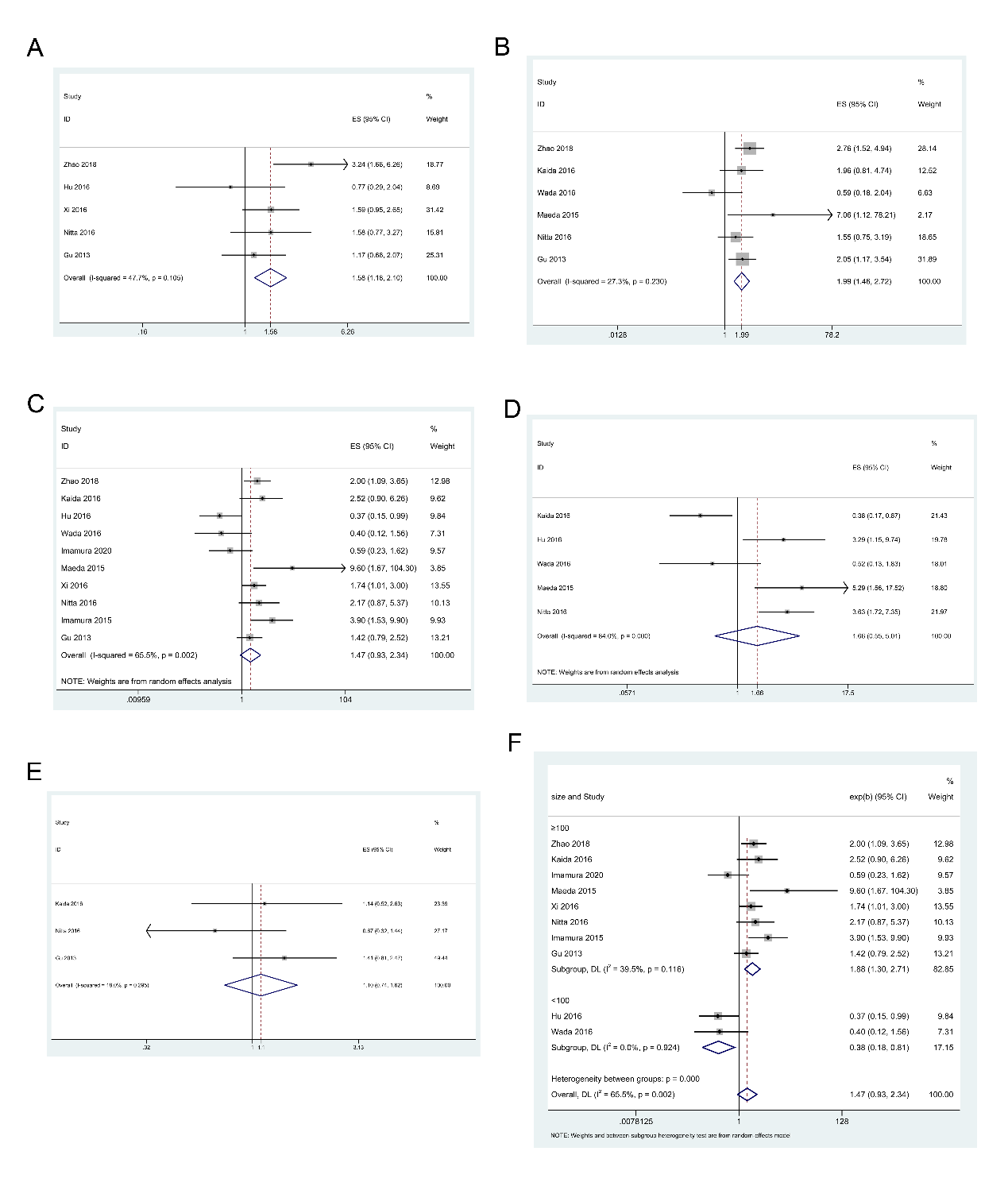


**Figure S1** Forest plot of studies evaluating the associations between the C5aR expression levels and clinicopathological features

A, tumor size; B, lymph node metastasis; C, tumor stage; D, vascular invasion; E, tumor differentiation; F, subgroup analysis for tumor stage


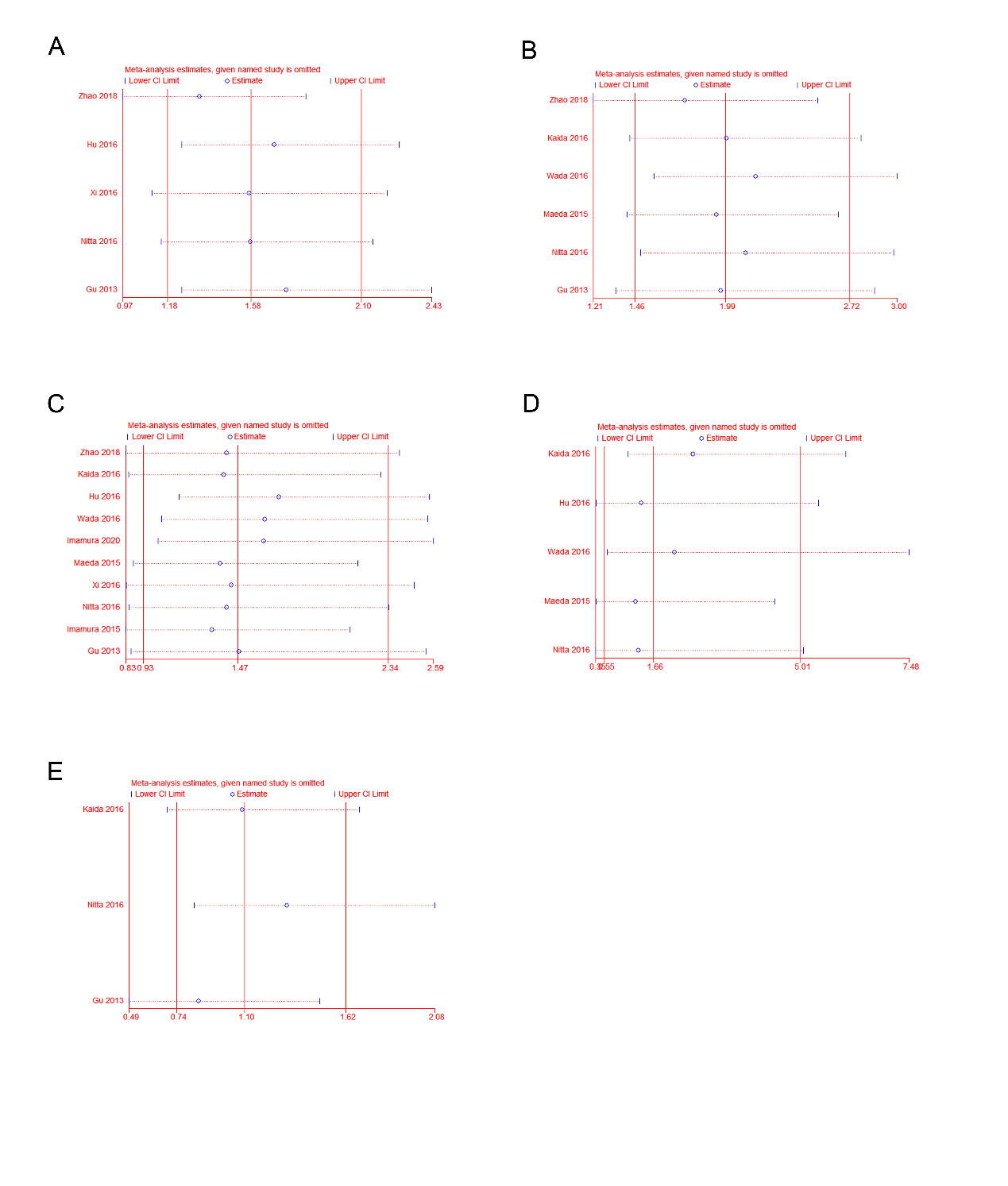


**Figure S2** Sensitivity analysis of studies evaluating the associations between the C5aR expression levels and clinicopathological features

A, tumor size; B, lymph node metastasis; C, tumor stage; D, vascular invasion; E, tumor differentiation


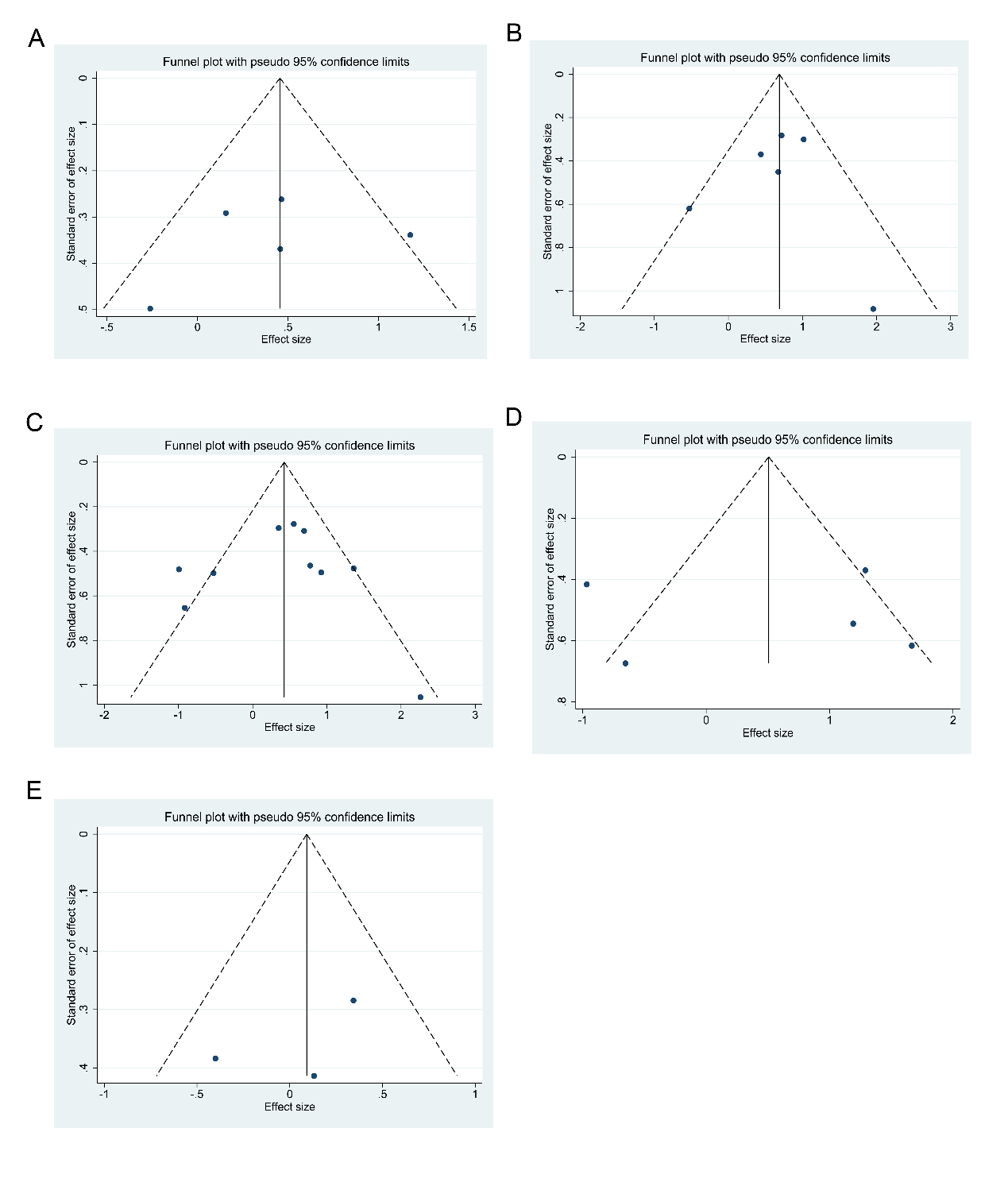


**Figure S3** Funnel plot for publication bias in this meta-analysis

A, tumor size; B, lymph node metastasis; C, tumor stage; D, vascular invasion; E, tumor differentiation
